# Effect of Substrate Orientation on Energy Storage in NaNbO_3_-Based Thin Films

**DOI:** 10.3390/nano16211405

**Published:** 2026-11-01

**Authors:** Huijuan Dong, Yongmei Xue, Jianwei Liu, Zhihong Liu, Zejun Wang

**Affiliations:** Department of Physics, Changzhi University, Changzhi 046011, China; 12022833@czc.edu.cn (Y.X.); ljw75_2002@163.com (J.L.); zhihongliu@hunnu.edu.cn (Z.L.); zejunwangcz@foxmail.com (Z.W.)

**Keywords:** NaNbO_3_-based thin films, substrate orientation, dielectric properties, energy storage performance

## Abstract

Substrate orientation offers a route to tailoring the dielectric and energy-storage properties of lead-free oxide thin films. In this work, 1.0 mol.% Mn-doped 0.96NaNbO_3_–0.04CaZrO_3_ (NNCZ–Mn) thin films were fabricated via a sol–gel method on (100)-, (110)-, and (111)-oriented Nb-doped SrTiO_3_ substrates and exhibited corresponding preferential out-of-plane orientations. The (111) film combined higher permittivity and lower leakage current with a larger recoverable polarization, yielding the best energy-storage performance among the three orientations. It achieved a recoverable energy density of approximately 23 J/cm^3^ with an efficiency of 65% at an electric field of 666.67 kV/cm and reached approximately 35 J/cm^3^ at 970 kV/cm with an efficiency of 48%. Its recoverable energy density also exhibited moderate thermal stability over 23–160 °C, good frequency stability over 400–2000 Hz, and good fatigue resistance after 10^6^ cycles. This study demonstrates the potential of substrate orientation control for improving solution-processed NaNbO_3_-based thin-film capacitors.

## 1. Introduction

Capacitive energy-storage technologies, including metal-oxide-based electrochemical supercapacitors and dielectric capacitors, have attracted considerable attention for electrical energy management applications [1,2]. Dielectric capacitors are essential for pulsed-power systems and electronic circuits because of their rapid charge–discharge response and high power capability [2,3]. Increasing the recoverable energy density while maintaining an acceptable energy-storage efficiency is essential for reducing device size and energy dissipation [3,4]. For nonlinear dielectric materials, energy storage depends on the polarization response and energy losses over the applied electric-field range, whereas hysteresis and electrical conduction contribute to energy loss. Therefore, a large recoverable polarization, low hysteresis loss, and low leakage current are beneficial for efficient energy storage [3,4]. Researchers have explored strategies including multilayer structures, compositional modification, structural disorder, and defect engineering to enhance dielectric energy-storage performance [5,6,7,8,9]. Among various dielectric materials, NaNbO_3_-based antiferroelectric thin films are promising candidates for high-energy-density capacitors owing to their large field-induced polarization changes, tunable phase structures, and environmental friendliness [10,11,12,13]. However, leakage current and hysteresis loss in pure NaNbO_3_ thin films may limit their energy-storage performance. Element doping modification has been demonstrated to be an effective strategy for tuning ferroelectric–antiferroelectric phase transitions [14,15,16], refining domain structures [7,17], reducing leakage current [18], and enhancing the energy-storage performance of NaNbO_3_-based materials [19]. Shimizu et al. [20] investigated the NaNbO_3_–CaZrO_3_ solid-solution system and demonstrated the stabilization of antiferroelectric behavior through compositional modification. In thin films, Shiraishi et al. [10] prepared CaZrO_3_-substituted NaNbO_3_ by chemical solution deposition and showed that substitution can improve the efficiency and thermal stability of energy storage. Mn modification offers a further means of tuning the electrical properties of this system [18]. Luo et al. [18] reported a recoverable energy density of approximately 19.64 J/cm^3^ and an efficiency of 64.5% at 833.3 kV/cm for 1.0 mol.% Mn-modified NaNbO_3_–CaZrO_3_ films, together with stable energy-storage behavior over 30–160 °C. These studies establish Mn-modified NaNbO_3_–CaZrO_3_ as a useful material system for investigating additional routes to improved thin-film performance.

In addition to composition, substrate orientation can influence the electrical properties of oxide thin films [21,22,23]. Orientation-dependent strain and domain responses in perovskite films have been investigated both experimentally and theoretically [21,22,24,25,26]. Substrate-related growth behaviors have likewise been explored in other material systems, such as Ta_3_N_5_ films grown on sapphire with a Ta_2_O_5_ seed layer [27]. For NaNbO_3_, Yamazoe et al. [28] investigated the effects of SrTiO_3_ substrate orientation on the surface morphology and dielectric and ferroelectric properties of thin films prepared by pulsed laser deposition. These findings confirm the correlation between substrate orientation and electrical responses. However, they do not directly establish how substrate orientation affects the energy-storage performance. A comparison at a fixed composition is therefore needed to clarify the relationships between substrate orientation, dielectric response, and recoverable energy storage.

In this work, 1.0 mol.% Mn-doped 0.96NaNbO_3_–0.04CaZrO_3_ (NNCZ–Mn) thin films were prepared on (100)-, (110)-, and (111)-oriented Nb-doped SrTiO_3_ (NSTO) substrates using a sol–gel method. The effects of substrate orientation on crystal structure, surface morphology, leakage current, dielectric properties, and polarization behavior were systematically investigated. The recoverable energy density and efficiency were evaluated from the polarization–electric-field loops, while the temperature stability, frequency dependence, and cycling endurance of the (111) sample were further examined. The results demonstrate that the (111) sample combines lower leakage current with higher permittivity and recoverable energy density. These findings clarify the relationships among substrate orientation, electrical responses, and energy-storage performance in NaNbO_3_-based thin films and highlight the necessity of further reducing energy loss to simultaneously achieve high recoverable energy density and high efficiency.

## 2. Materials and Methods

### 2.1. Preparation of Thin Films

The 1.0 mol.% Mn-doped 0.96NaNbO_3_–0.04CaZrO_3_ (NNCZ–Mn) thin films were prepared by a sol–gel route on (100)-, (110)-, and (111)-oriented 0.7% Nb-doped SrTiO_3_ (NSTO) single-crystal substrates [10,18]. Sodium acetate (CH_3_COONa), niobium ethoxide [Nb(OC_2_H_5_)_5_], calcium acetate monohydrate [Ca(CH_3_COO)_2_·H_2_O], zirconium butoxide [Zr(OC_4_H_9_)_4_], and manganese acetate tetrahydrate [Mn(CH_3_COO)_2_·4H_2_O] were used as metal precursors. Acetic acid, 2-methoxyethanol, and deionized water were used as solvents, while acetylacetone was added as a chelating agent. The precursors were mixed in stoichiometric ratios and dissolved to obtain a solution with a concentration of 0.5 mol/L. Solution preparation was performed in a glove box under a dry nitrogen atmosphere to prevent uncontrolled hydrolysis of niobium ethoxide. A 5 mol.% excess of sodium was added to compensate for sodium loss during thermal treatment.

The precursor solution was spin-coated onto the NSTO substrates at 5500 rpm for 30 s. Each deposited layer was dried at 120 °C for 10 min to evaporate the solvents and subsequently pyrolyzed at 400 °C for 5 min to remove residual organic components. The samples were then cooled to room temperature. The spin-coating and thermal treatments were repeated three times to obtain films with a thickness of approximately 300 nm. Finally, the films were crystallized at 680 °C for 10 min.

### 2.2. Structural and Electrical Characterization

The crystalline structure of the films was characterized by X-ray diffraction (XRD, Bruker D8, Karlsruhe, Germany) using Cu Kα radiation. The surface morphology was examined by atomic force microscopy (AFM, MFP-3D, Asylum Research, Santa Barbara, CA, USA) over a scanning area of 2 × 2 μm^2^, and the root-mean-square surface roughness was determined from the AFM images.

For electrical measurements, circular Pt top electrodes with a diameter of 0.2 mm were deposited by sputtering through a shadow mask, with the conducting NSTO substrates serving as the bottom electrodes. Polarization–electric-field (*P*–*E*) hysteresis loops were measured using a Radiant Precision workstation at a frequency of 1 kHz. Leakage-current characteristics were measured using a Keithley 6487 instrument, and the dielectric constant and loss tangent were measured using an Agilent E4980 LCR meter over 10^3^–10^6^ Hz.

### 2.3. Energy-Storage Calculations

The recoverable energy density (*W*_*r*_) and energy-storage efficiency (*η*) were evaluated from the measured *P*–*E* hysteresis loops [3,4]. *W*_*r*_ was calculated by integrating the discharge branch from the remanent polarization (*P*_*r*_) to the maximum polarization (*P*_*max*_):

(1) $$W_{r}=\int_{P_{r}}^{P_{max}}\mathrm{EdP}$$

The total energy density was determined by integrating the charging branch from zero polarization to *P*_*max*_:

(2) $$W_{\mathrm{tot}}=\int_{0}^{P_{max}}\mathrm{EdP}$$

The energy loss was calculated as the difference between the total and recoverable energy densities:

*W_loss_* = *W_tot_* − *W_r_* (3)

The energy-storage efficiency was therefore expressed as

(4) $$\eta =\frac{W_{r}}{W_{\mathrm{tot}}}\times 100\%$$

Here, *E* and *P* denote the electric field and polarization, respectively.

## 3. Results and Discussion

**Figure 1:**
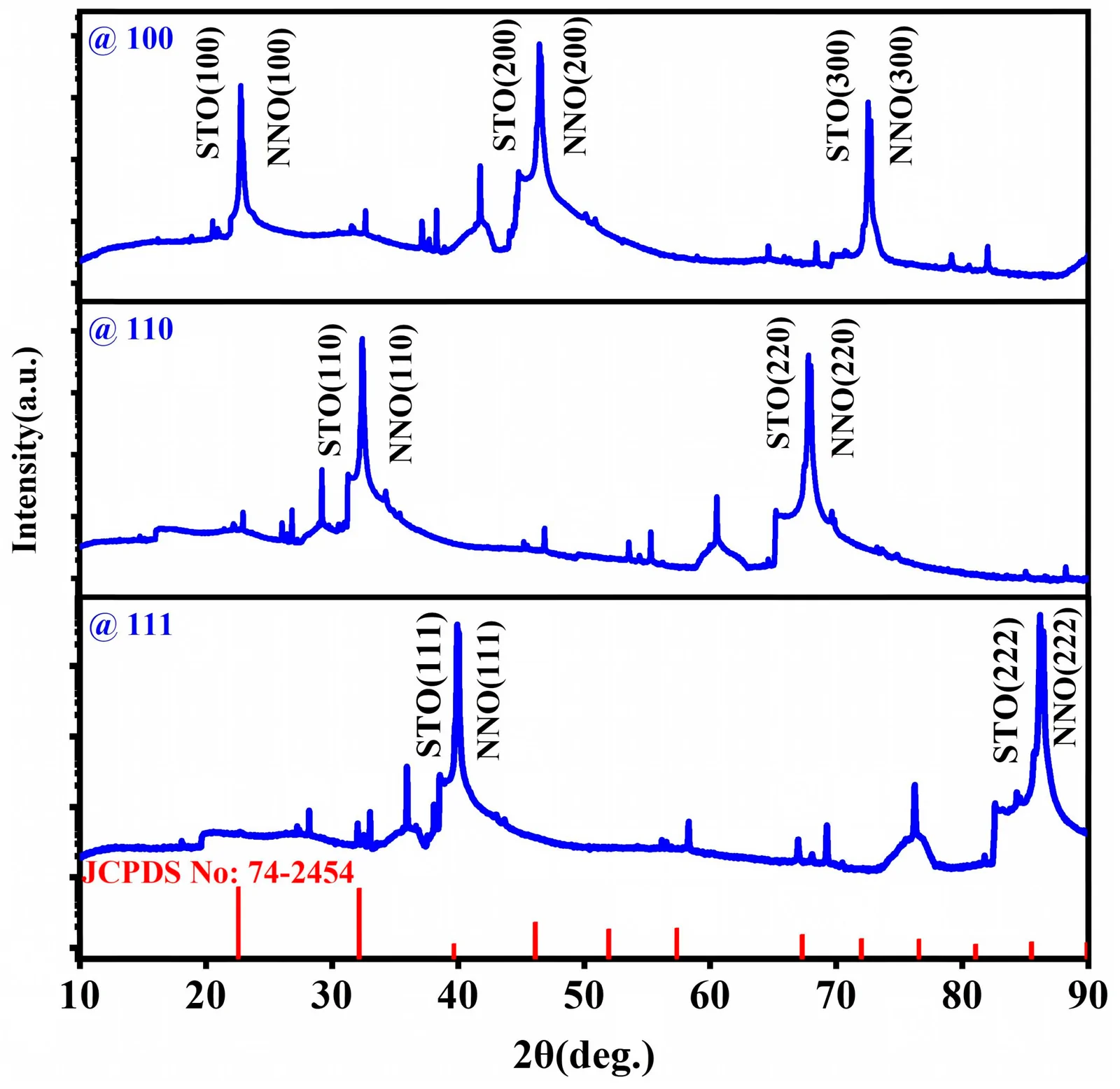
X-ray diffraction patterns of NNCZ–Mn films on (100)-, (110)-, and (111)-oriented NSTO substrates. The labels “@100”, “@110”, and “@111” denote films grown on (100)-, (110)-, and (111)-oriented NSTO substrates, respectively.

Figure 1 presents the X-ray diffraction patterns of NNCZ–Mn thin films deposited on (100)-, (110)-, and (111)-oriented NSTO substrates. The (100), (200), and (300) reflections of the film on (100) NSTO appeared near the corresponding substrate reflections. Similarly, the (110) and (220) reflections of the film on (110) NSTO, and the (111) and (222) reflections of the film on (111) NSTO, were observed close to those of the respective substrates. The film peaks were close to and partially overlapped with the substrate peaks because NaNbO_3_-based films and NSTO possess similar lattice parameters of approximately 3.90 Å. According to Bragg’s law, their comparable interplanar spacings give rise to closely spaced diffraction angles [18,28]. The competing lattice contraction and expansion associated with Ca^2+^ and Zr^4+^ substitution may further limit the change in the lattice dimensions of NNCZ films, as discussed by Luo et al. [18]. Although the partial overlap between film and substrate reflection limits the measurement of the film’s lattice parameters, the observed diffraction patterns indicate that the NNCZ–Mn films exhibit preferential (100), (110), and (111) out-of-plane orientations on the corresponding NSTO substrates.

**Figure 2:**
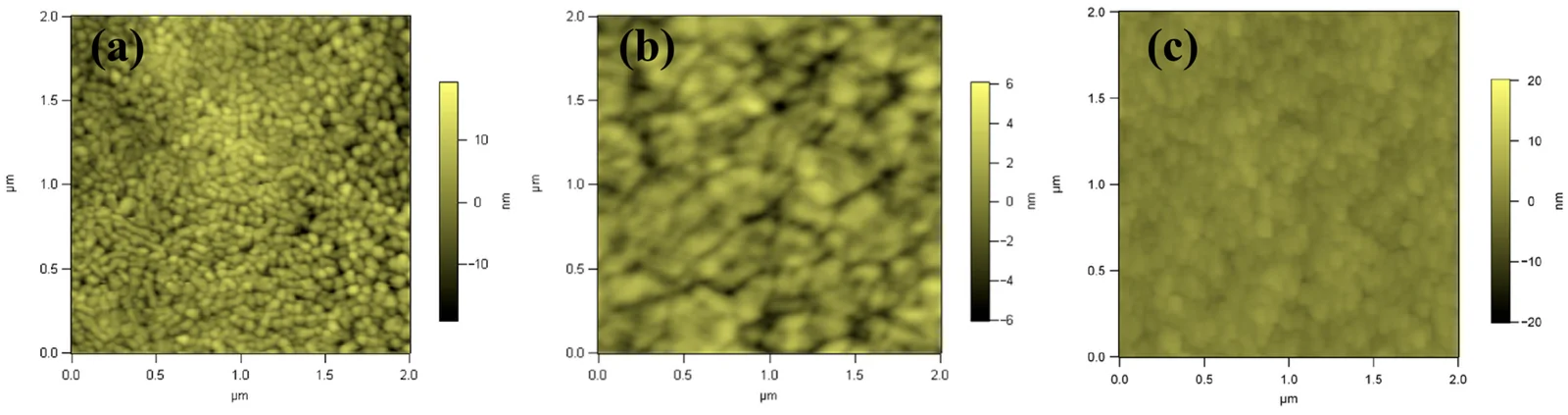
AFM surface topography of NNCZ–Mn films on (**a**) (100), (**b**) (110), and (**c**) (111) NSTO substrates.

The surface morphologies of NNCZ–Mn films deposited on differently oriented NSTO substrates were examined by AFM over a scanning area of 2 × 2 μm^2^, as shown in Figure 2. The films exhibit distinct surface morphologies depending on the substrate orientation. The (100) sample exhibits relatively fine surface features, the (110) film shows broader features, and the (111) film has a fine granular morphology. All films show uniformly dense surfaces without obvious cracks or voids. The corresponding RMS surface roughness values are approximately 2.82, 1.41, and 1.29 nm for the (100), (110), and (111) films, respectively. The surface morphology of the films is influenced by thermal processing and surface/interface energetics. In this sol–gel route, the thermal treatment sequence comprises drying at 120 °C, pyrolysis at 400 °C, and crystallization at 680 °C. The three films were compared under the same processing conditions. Therefore, the observed differences in roughness reflect the orientation dependence of the films, which is consistent with the process sensitivity of NaNbO_3_-based films prepared by the solution method as previously reported [13,18]. Among the three orientations, the (111)-oriented film has the lowest surface roughness, which may contribute to lower leakage current and dielectric loss.

**Figure 3:**
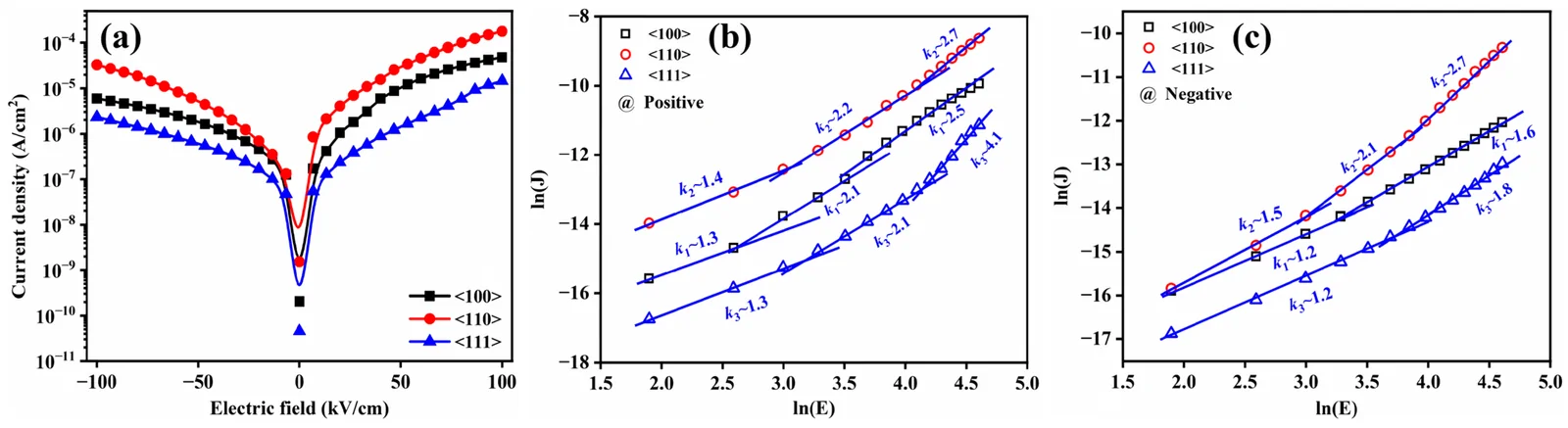
Leakage-current characteristics of NNCZ–Mn films on differently oriented NSTO substrates: (**a**) Current density as a function of applied electric field. Double-logarithmic current–field fits under (**b**) positive and (**c**) negative bias. The labels @ Positive and @ Negative represent the positive bias region and negative bias region, respectively.

Figure 3a shows the room-temperature leakage-current density of NNCZ–Mn films grown on differently oriented NSTO substrates as a function of electric field. The current density increases with increasing electric-field magnitude for all three samples. Over the measured field range, the (111) sample exhibits the lowest leakage-current density, followed by the (100) sample, whereas the (110) sample shows the highest leakage. Subsequently, the J–E curves were fitted using conduction models for dielectric thin films to further analyze the leakage behavior. The double-logarithmic fits under positive and negative bias are shown in Figure 3b and Figure 3c, respectively. The slopes were evaluated using the power-law relationship *J*∝*E*^n^. Under positive bias, the low-field slopes are approximately 1.3–1.4, indicating dominant Ohmic conduction. With increasing electric field, the slopes approach 2.1–2.2, showing a nearly quadratic current–field relationship (*J*∝*E*^2^), consistent with Child’s law for space-charge-limited conduction. In this region, the injected charge carriers may gradually fill the available trap states. At higher fields, the slopes increase further to approximately 2.5, 2.7, and 4.1 for the (100)-, (110)-, and (111)-oriented films, respectively. This increasingly nonlinear behavior may be associated with trap filling and a possible transition toward the trap-filled-limit regime.

Under negative bias, the slope in low electric fields is approximately 1.2–1.5, indicating that the ohmic contribution dominates for all three films. As the electric field increases, the slopes of the (100)-oriented and (111)-oriented films rise to approximately 1.6 and 1.8, respectively, indicating a gradual deviation from ohmic conduction. For the (110)-oriented film, the slope increases to approximately 2.1 under moderate electric fields, which is consistent with Child’s law governing space-charge-limited conduction. At even higher electric fields, the slope further increases to approximately 2.7, suggesting a possible contribution from a trap-filling process [29].

Compared to a negative bias, positive bias induces a clearer transition from near-ohmic behavior to the near-quadratic region, followed by a steeper slope at higher electric fields. This difference is especially pronounced in (111)-oriented films, whose high-field slope reaches approximately 4.1 under positive bias but only 1.8 under negative bias. The leakage-current curves also exhibit asymmetry, with higher current densities under positive bias at comparable voltage magnitudes. This asymmetry may arise from the different electrode materials and interface characteristics at the Pt/NNCZ–Mn and NNCZ–Mn/NSTO interfaces. The resulting differences in carrier-injection barriers can lead to unequal charge injection under opposite voltage polarities [29,30,31].

**Figure 4:**
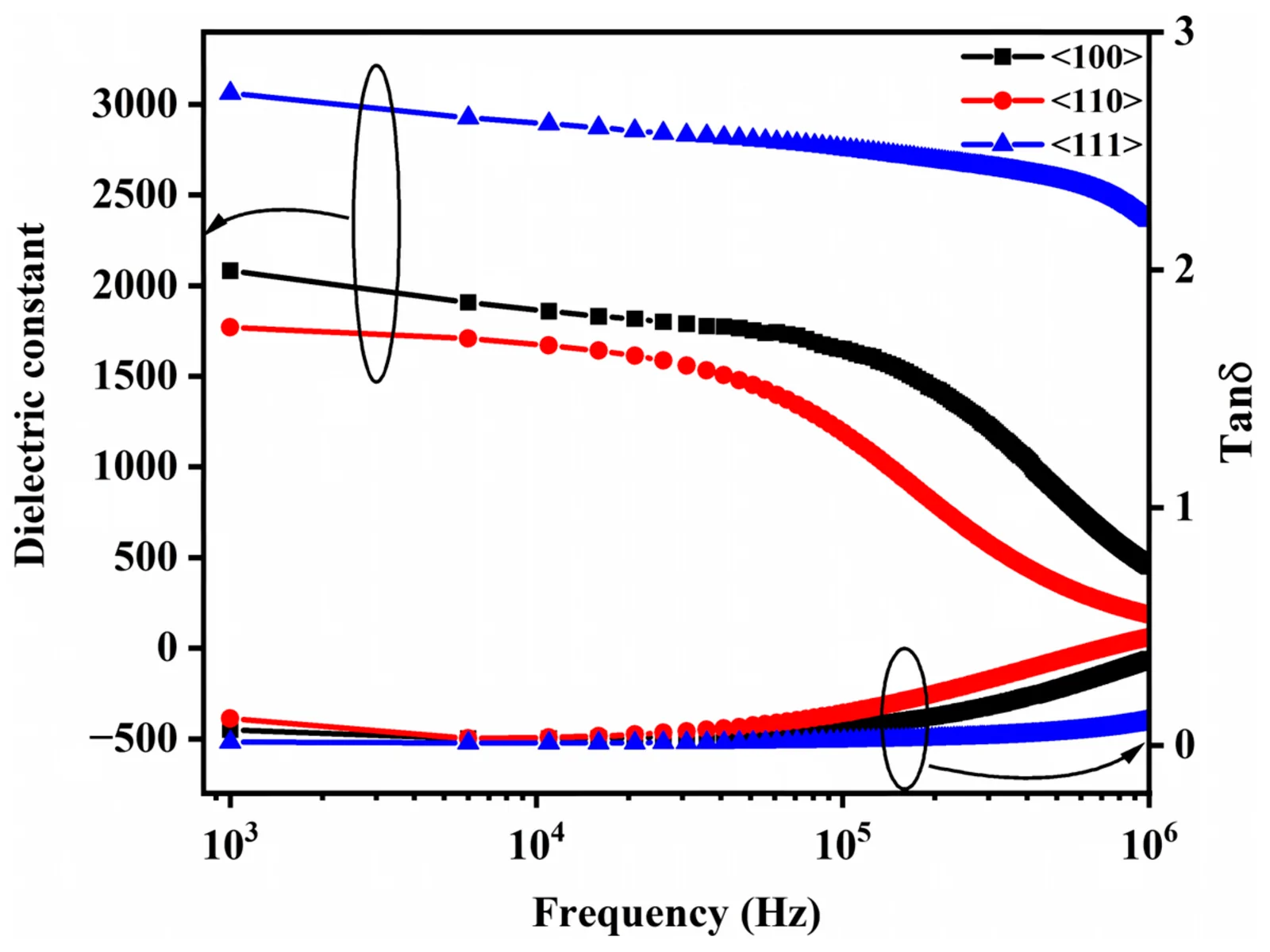
Frequency-dependent relative permittivity (three curves surrounded by black circles with left arrows) and dielectric loss tangent, tan δ (three curves surrounded by black circles with right arrows), for the three substrate orientations.

The dielectric response of NNCZ–Mn films grown on differently oriented NSTO substrates was characterized at room temperature over a frequency range of 10^3^–10^6^ Hz, as shown in Figure 4. The dielectric constant follows the order (111) > (100) > (110). At approximately 10 kHz, the dielectric constants are about 2900, 1900, and 1700 for the (111), (100), and (110) samples, respectively. As reported by Yamazoe et al. [28], the dielectric response of NaNbO_3_ films depends on crystallographic orientation and the direction of the applied field relative to the polarization. The different dielectric constants observed here may therefore reflect orientation-dependent polarization responses and domain configurations [21,32,33].

The relative dielectric constant decreases with increasing frequency, particularly for the (100) and (110) samples above approximately 10^5^ Hz. This dispersion may reflect a reduced contribution from slower polarization processes, including interfacial polarization and domain-wall motion [18,32]. Meanwhile, tan δ increases toward the high-frequency region, with the (111) sample exhibiting a comparatively smaller increase. These results indicate that the (111) sample combines a higher dielectric constant with lower dielectric loss over the measured frequency range.

**Figure 5:**
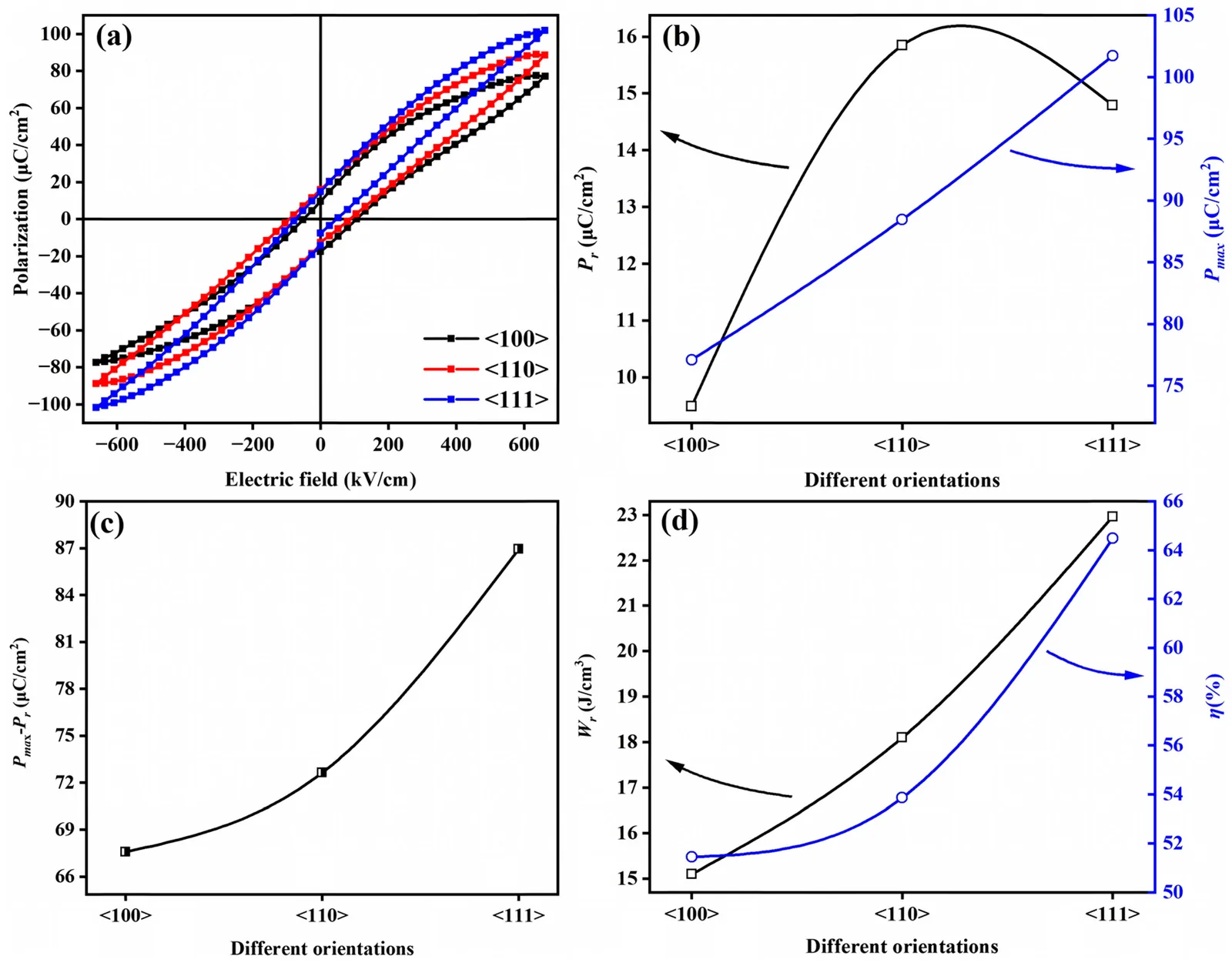
Polarization response and energy-storage performance for the three substrate orientations: (**a**) *P*–*E* loops, (**b**) remanent polarization (black squares with left arrow) and maximum polarization (blue circles with right arrow), (**c**) *P*_ma*x*_ − *P*_r_, and (**d**) recoverable energy density (black squares with left arrow) and efficiency (blue circles with right arrow).

To examine the effect of substrate orientation on polarization and energy-storage behavior, the *P*–*E* loops of NNCZ–Mn films were measured at the same electric field of 666.67 kV/cm and frequency of 1 kHz, as shown in Figure 5a. The maximum polarization (*P*_max_) increases from approximately 77 to 89 and 102 μC/cm^2^ for the (100), (110), and (111) samples, respectively. In contrast, the remanent polarization (*P*_r_) increases from approximately 9.5 μC/cm^2^ for (100) to 15.8 μC/cm^2^ for (110), then decreases slightly to 14.8 μC/cm^2^ for (111) (Figure 5b). Consequently, the (111) sample exhibits the largest polarization difference, *P*_max_−*P*_r_, of approximately 87 μC/cm^2^ (Figure 5c).

The recoverable energy density (*W*_r_) and efficiency (*η*), calculated from the *P*–*E* loops, are summarized in Figure 5d. Both *W*_r_ and *η* increase from (100) through (110) to (111), with *W*_r_ increasing from approximately 15 to 18 and 23 J/cm^3^, and *η* increasing from approximately 51% to 54% and 65%, respectively. The improved energy-storage response of the (111) sample is associated with its larger recoverable polarization interval and more favorable charging–discharging energy balance [3,34]. These results demonstrate that substrate orientation influences the polarization and energy-storage properties, with the (111) sample providing the highest *W*_r_ and *η*.

**Figure 6:**
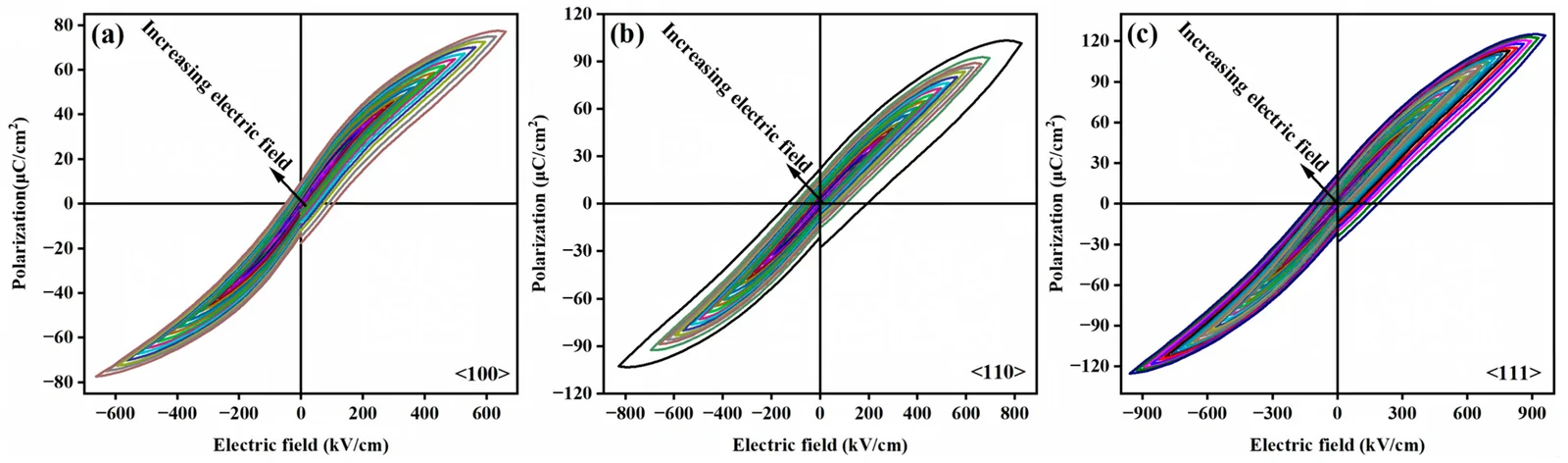
*P*–*E* loops for NNCZ–Mn films on (**a**) (100), (**b**) (110), and (**c**) (111) NSTO. The different colors represent *P*–*E* loops measured under different electric fields, and the arrows indicate the direction of increasing electric field amplitude.

Figure 6 shows the *P*–*E* hysteresis loops of NNCZ–Mn films on (100)-, (110)-, and (111)-oriented NSTO substrates measured at progressively increasing electric fields. The polarization increases with an increasing applied field for all three samples. At their respective highest displayed fields, the maximum polarization reaches approximately 80, 105, and 125 μC/cm^2^ for the (100), (110), and (111) samples, respectively. The (111) sample exhibits the widest measured field range and the largest polarization response. These results indicate that substrate orientation influences the high-field polarization behavior of NNCZ–Mn films, with the (111) sample offering a larger accessible polarization range for energy storage.

**Figure 7:**
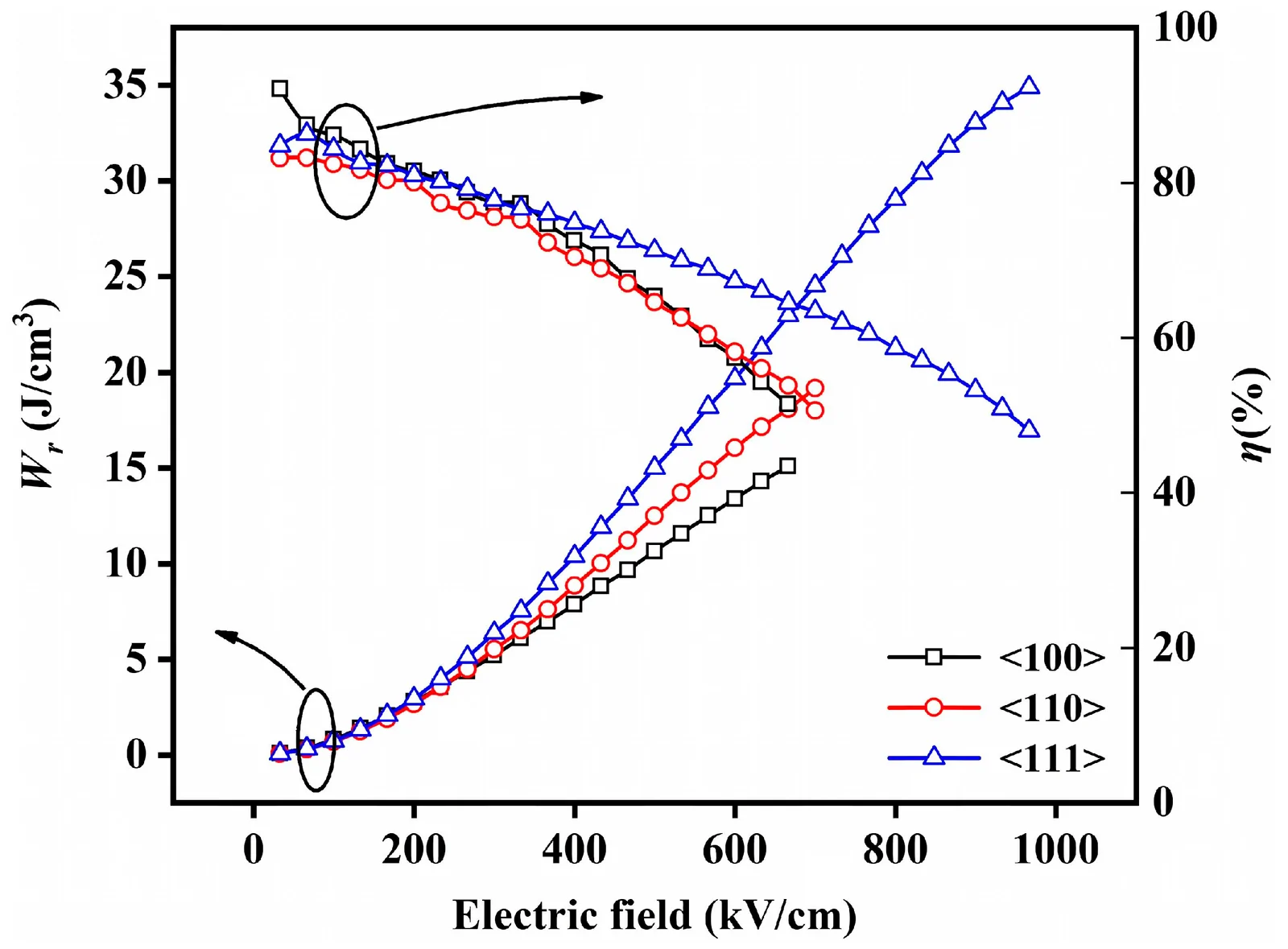
Electric-field dependence of *W*_r_ (three curves surrounded by black circles with left arrows) and *η* (three curves surrounded by black circles with right arrows) for NNCZ–Mn films on (100), (110), and (111) NSTO at room temperature.

The electric-field dependence of the recoverable energy density (*W*_r_) and efficiency (*η*) of NNCZ–Mn films on differently oriented NSTO substrates is shown in Figure 7. *W*_r_ increases with an increasing electric field for all three samples, whereas *η* generally decreases, indicating that dissipation gradually increases at higher electric fields. At comparable fields in the higher-field region, the (111) sample exhibits a larger *W*_r_ than the (100) and (110) samples.

At their respective highest fields, *W*_r_ reaches approximately 15, 19, and 35 J/cm^3^ for the (100), (110), and (111) films, respectively. The (111) sample extends to approximately 970 kV/cm, where its efficiency is approximately 48%. Its higher maximum energy density therefore benefits from both a favorable polarization response and a wider measured field range. The (111) substrate provides the most favorable energy-density response among the three orientations, while the decrease in efficiency highlights the trade-off between recoverable energy and high-field loss.

**Figure 8:**
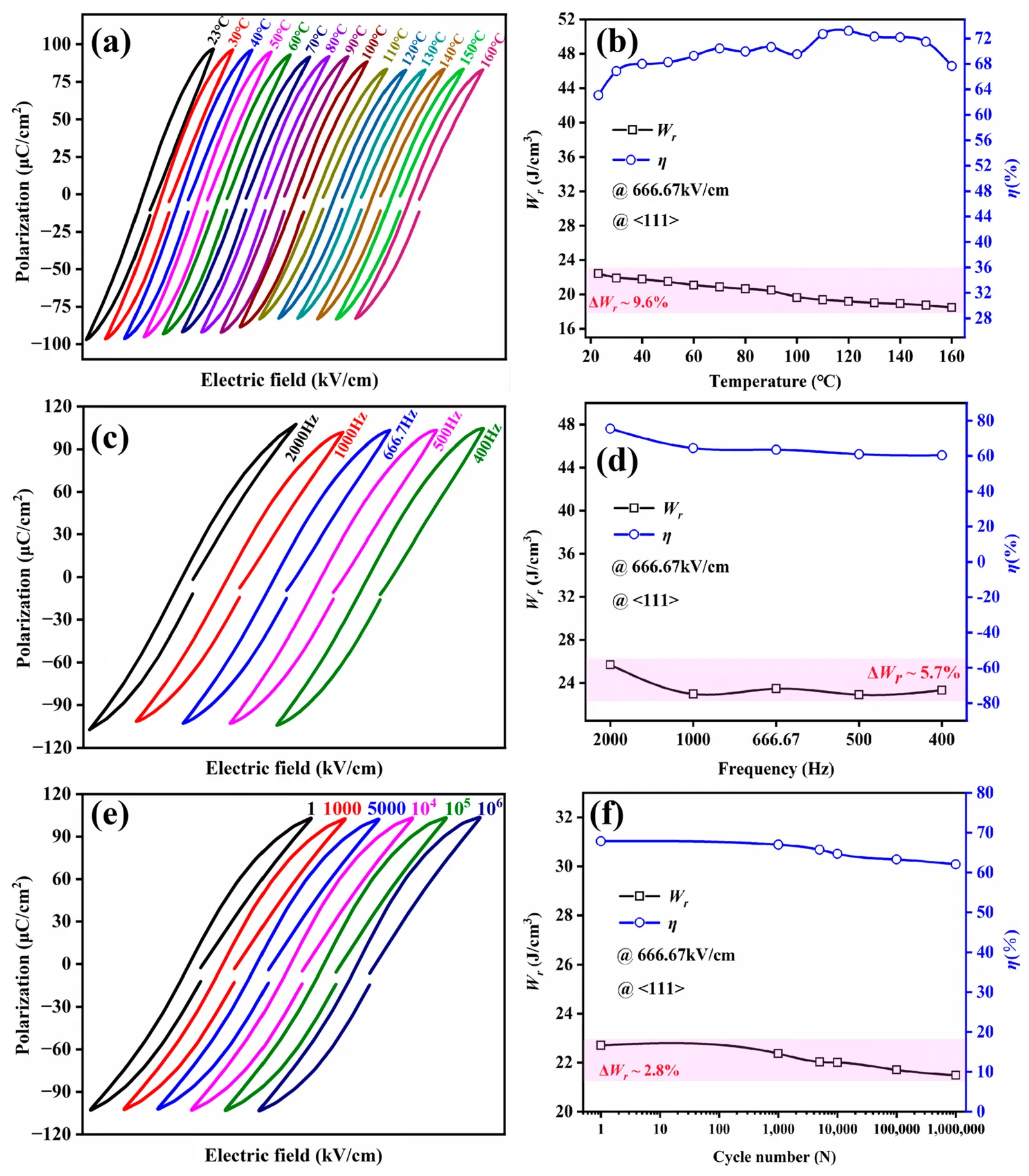
Temperature, frequency, and cycling dependence of the energy-storage properties for the (111)-oriented NNCZ–Mn film at a field of 666.67 kV/cm: (**a**,**c**,**e**) *P*–*E* loops. (**b**,**d**,**f**) *W*_r_ and *η* values. In (**b**,**d**,**f**), the symbol “@” represents the test electric field and substrate orientation of the film.

Based on the excellent energy-storage performance of (111)-oriented NNCZ–Mn films, their temperature stability, frequency dependence, and cycling endurance were further investigated, as shown in Figure 8. Under an electric field of 666.67 kV/cm, *P*-*E* loops were measured over a temperature range of 23–160 °C, and the corresponding *W*_r_ and *η* values were calculated, as shown in Figure 8a,b. As the temperature increases, *W*_r_ gradually decreases, with a variation of approximately 9.6% in Figure 8b. Meanwhile, *η* increases from approximately 63% near room temperature and remains around 63–73% over most of the higher-temperature range. Figure 8c,d shows the frequency-dependent response over 400–2000 Hz. The variation in *W*_r_ is approximately 5.7%, while *η* increases from approximately 60% at 400 Hz to 75% at 2000 Hz. Finally, fatigue-dependent *P*-*E* hysteresis loops were measured at room temperature for up to 10^6^ electrical cycles, and the *W*_r_ and *η* values were calculated, as shown in Figure 8e,f. The amplitude of *W*_r_ fluctuations was approximately 2.8%. The efficiency decreased from approximately 68% to 62%. These results suggest that the recoverable energy density of the (111) sample exhibits reasonable thermal stability, good frequency stability, and fatigue stability.

Table 1 compares the present (111)-oriented film in this study with representative lead-free films prepared via sol–gel processing or chemical solution deposition. This film achieved a competitive recoverable energy density of 35 J/cm^3^ under a relatively low applied electric field of 970 kV/cm. However, its efficiency of 48% is lower than that of many previously reported films, highlighting the need for further optimization to reduce energy losses and improve efficiency.

## 4. Conclusions

NNCZ–Mn thin films with preferential (100), (110), and (111) out-of-plane orientations were prepared on the corresponding NSTO substrates by a sol–gel method. Substrate orientation affected the surface morphology, leakage current, dielectric response, and polarization behavior. The (111) sample exhibited the lowest surface roughness and leakage-current density, as well as the highest relative permittivity. Under an electric field of 666.67 kV/cm, its recoverable energy density and efficiency reached approximately 23 J/cm^3^ and 65%, respectively, exceeding those of the (100) and (110) samples. Increasing the field to approximately 970 kV/cm raised the recoverable energy density to approximately 35 J/cm^3^, while the efficiency decreased to 48%, indicating a trade-off between energy density and high-field loss. The positive- and negative-bias leakage fits suggest field-dependent transport involving approximately Ohmic and possible space-charge/trap-related contributions. The (111) film also maintained relatively stable recoverable energy density over 23–160 °C, 400–2000 Hz, and 1–10^6^ cycles. These findings demonstrate that substrate orientation is a means of improving the energy-storage response, while further optimization is required to reduce energy losses and achieve both high *W*_r_ and high efficiency.

## Figures and Tables

**Table 1:** Energy-storage performance of representative lead-free thin films prepared by sol–gel processing or chemical solution deposition (CSD).

Thin-Film Material	Method	*E* (kV/cm)	*W*_r_ (J/cm^3^)	*η* (%)	Ref.
BaZr_0.2_Ti_0.8_O_3_–0.02MnO_2_	Sol–gel	3700	32.3	62	[35]
Ba_0.85_Sr_0.15_TiO_3_	Sol–gel	2000	7.19	64	[36]
Mn-modified 0.80NaNbO_3_–0.20Bi(Mg_2/3_Nb_1/3_)O_3_	CSD	2450	37	80	[13]
Mn-modified 0.85Bi_0.5_Na_0.5_TiO_3_–0.15Bi(Mg_2/3_Nb_1/3_)O_3_	CSD	2700	61	70	[37]
0.5(Bi_0.5_Na_0.5_)_0.94_Ba_0.06_TiO_3_–0.5Bi(Mg_0.5_Ti_0.5_)O_3_	Sol–gel	2149	34.36	56.63	[38]
NNCZ–Mn, (111) orientation	Sol–gel	970	35	48	This work

## Data Availability

The original contributions presented in this study are included in the article. Further inquiries can be directed to the corresponding author.
